# Pleistocene footprints show intensive use of lake margin habitats by *Homo erectus* groups

**DOI:** 10.1038/srep26374

**Published:** 2016-05-20

**Authors:** Neil T. Roach, Kevin G. Hatala, Kelly R. Ostrofsky, Brian Villmoare, Jonathan S. Reeves, Andrew Du, David R. Braun, John W. K. Harris, Anna K. Behrensmeyer, Brian G. Richmond

**Affiliations:** 1 Department of Human Evolutionary Biology, Harvard University, Cambridge, Massachusetts 02138, USA; 2 Division of Anthropology, American Museum of Natural History, New York, New York 10024, USA; 3 Department of Human Evolution, Max Planck Institute for Evolutionary Anthropology, Leipzig 04103, Germany; 4 Center for the Advanced Study of Human Paleobiology, The George Washington University, Washington, District of Columbia 20052, USA; 5 Department of Anthropology, University of Nevada - Las Vegas, Las Vegas, Nevada 89154, USA; 6 Department of Anthropology, Rutgers University, New Brunswick, New Jersey 08901, USA; 7 Department of Paleobiology, MRC: NHB 121, National Museum of Natural History, Smithsonian Institution, Washington, District of Columbia 20013-7012, USA

## Abstract

Reconstructing hominin paleoecology is critical for understanding our ancestors’ diets, social organizations and interactions with other animals. Most paleoecological models lack fine-scale resolution due to fossil hominin scarcity and the time-averaged accumulation of faunal assemblages. Here we present data from 481 fossil tracks from northwestern Kenya, including 97 hominin footprints attributed to *Homo erectus*. These tracks are found in multiple sedimentary layers spanning approximately 20 thousand years. Taphonomic experiments show that each of these trackways represents minutes to no more than a few days in the lives of the individuals moving across these paleolandscapes. The geology and associated vertebrate fauna place these tracks in a deltaic setting, near a lakeshore bordered by open grasslands. Hominin footprints are disproportionately abundant in this lake margin environment, relative to hominin skeletal fossil frequency in the same deposits. Accounting for preservation bias, this abundance of hominin footprints indicates repeated use of lakeshore habitats by *Homo erectus*. Clusters of very large prints moving in the same direction further suggest these hominins traversed this lakeshore in multi-male groups. Such reliance on near water environments, and possibly aquatic-linked foods, may have influenced hominin foraging behavior and migratory routes across and out of Africa.

Understanding the selective forces that drove human evolutionary history requires integrating different scales of ecological information. Theories regarding how global climate change affects local environmental conditions and alters hominin land use and behavior feature prominently in paleoanthropology^1^,^2^,^3^,^4^. Unfortunately, the fossil record typically lacks the precision to address questions of hominin paleoecology at fine temporal and spatial scales because fossil assemblages comingle remains from many thousands of years and multiple habitats. Such issues of scale make it difficult to separate biological variation within a single habitat from variation across neighboring or consecutive habitats.

Fossil tracks provide a unique opportunity to closely examine species’ habitat associations and behavior over very short time intervals. Tracks can form anywhere on a landscape with fine-grained sediments, typically when wetted by surface water or rainfall, increasing the cohesion of the substrate. Temporary preservation of tracks depends on how well the definition of the track is maintained as the sediment dries and hardens^5^. However, long-term preservation is largely dependent on how quickly the track-bearing surface is covered by additional, protective sediments^6^,^7^. Given that trackways are ephemeral and deteriorate quickly after formation^5^,^8^, groups of tracks in similar preservational states are assumed to be made either simultaneously or in very close temporal proximity to each other, possibly representing a social group. Trackways with sub-parallel alignment and minimal overlap and intersection provide further support for instantaneous track formation and group movement^9^,^10^, but may alternatively reflect the repeated movements of lone individuals around geographic constraints, such as a game trail, cliff or river^5^,^8^,^11^,^12^. Although track orientations have been frequently argued to document social structure and group movement in dinosaurs^5^,^9^,^13^,^14^,^15^,^16^,^17^, elephants^10^ and early hominins^18^,^19^, until recently only a handful of hominin footprints were known from the later, Pleistocene fossil record^18^,^20^.

## Geology and Depositional Context

**Figure 1:**
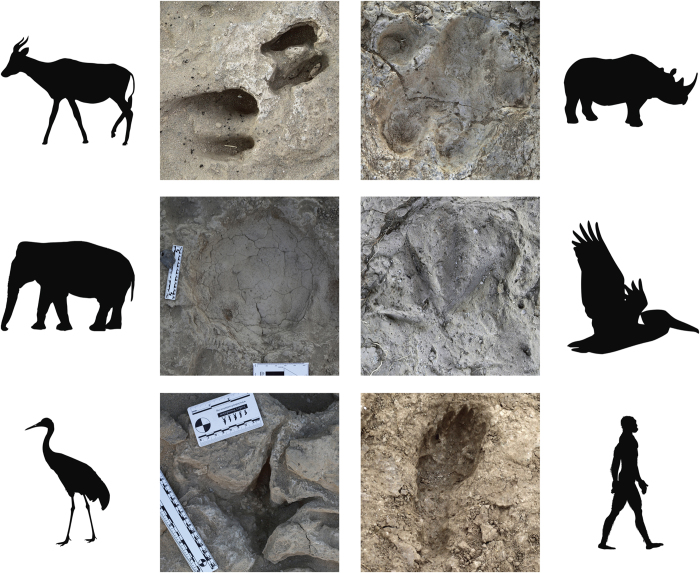
Photographs of 1.5 Ma tracks recovered near Ileret, Kenya. Clockwise from upper right: White rhinoceros (*Ceratotherium simum*), pelican (*Pelecanus*), hominin (putative *Homo erectus*), large wading bird (Ciconiidae or Gruidae), elephant (*Elephas* or *Loxodonta*) and medium sized bovid. Photos: N. Roach/K. Hatala. Silhouettes: www.phylopic.org, elephant by T. Michael Keesey (http://creativecommons.org/licenses/by/3.0).

**Figure 2:**
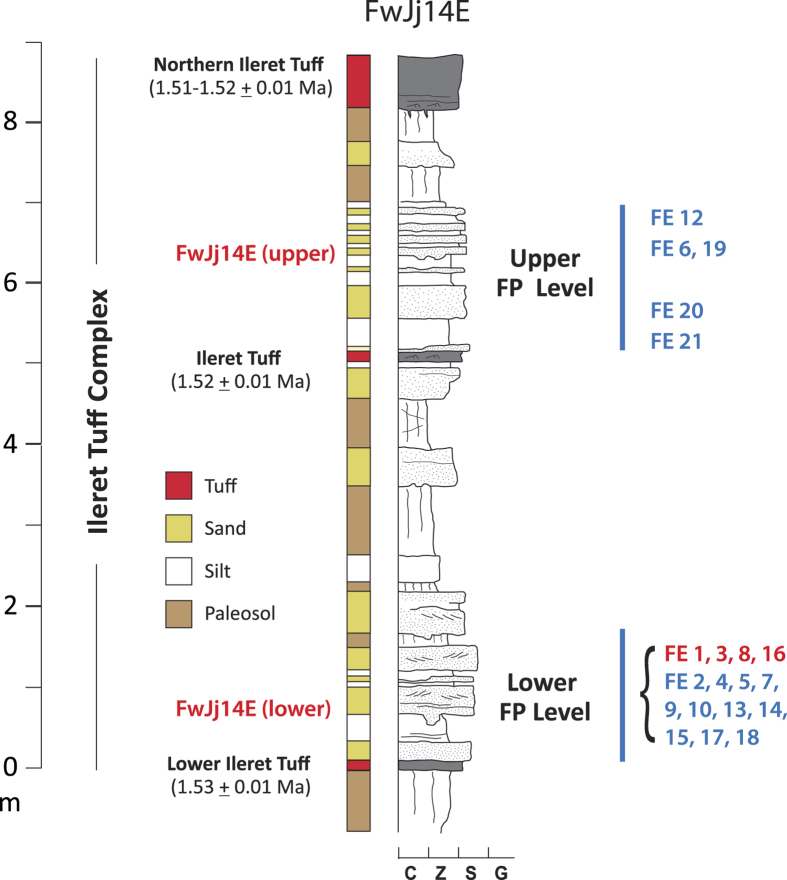
Representative stratigraphic column for the ITC, Okote member, Koobi Fora Formation based on the sequence at FwJj14E. Three ^40^Ar/^39^Ar dated tuffs bracket the sequence. Well-stratified, interbedded silt and fine sand units represent periods of active deposition on a delta margin, and the paleosols represent temporary, emergent land surfaces, all occurring within an estimated time interval of ~20,000 years. Zones containing preserved tracks are shown with blue bars. The lower cluster of sites occurs at multiple levels along 800 m of outcrop within the indicated stratigraphic interval. Note that zones without track surfaces show significant paleosol development and sediment modification. Random test square and targeted excavation levels are noted on the right (red sites contain hominin prints; blue sites do not).

We report here on 481 identifiable fossil tracks (Fig. 1), including 97 hominin footprints, found near the town of Ileret in northwestern Kenya. A small assemblage of hominin and other animal tracks was initially discovered in 2006^21^. The excavation of this site has continued over the past 9 years, and new excavations were conducted in 2013–2014 at three additional targeted localities where hominin prints were also found. Twenty randomly selected test squares also were excavated, totaling 114 m^2^ of uncovered track surface. These surfaces are located within the Okote Member of the Koobi Fora Formation and are tightly time bracketed between fluvially reworked volcanic tuffs. The Northern Ileret Tuff caps the sequence and is radiometrically dated to 1.51–1.52 Ma, while the underlying Lower Ileret Tuff is dated to 1.53 Ma^22^,^23^. Between these tuffs is ~8.5 m of massive and laminated silts interspersed with fine grained, stratified and cross-stratified sands (Fig. 2). This complex is divided near the middle by the Ileret Tuff, dated to 1.52 Ma^21^.

**Figure 3:**
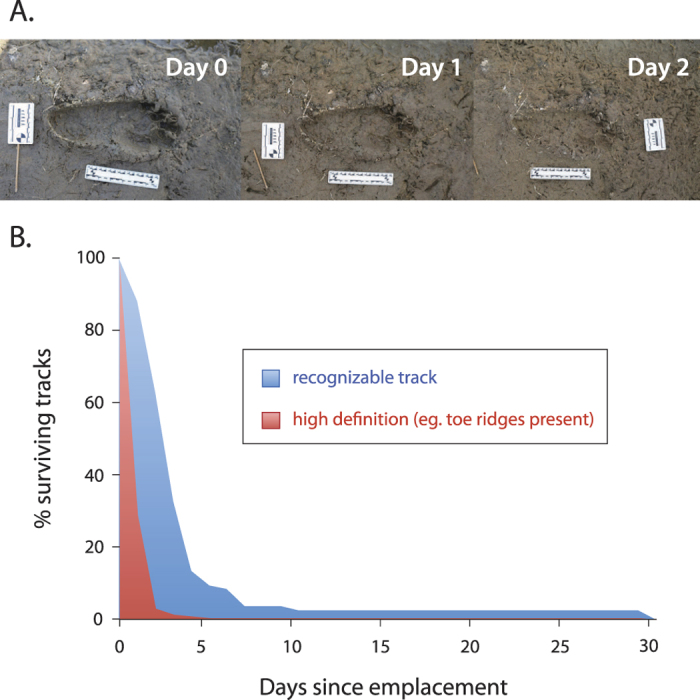
Track duration. Taphonomy experiments show human footprints retained high definition features (in red) an average of only 1.3 days (**A**), with 0.5% of tracks preserving these features longer than 4 days (1 track retained toe ridges for at least 1 month). Notably, tracks formed in deeper muds, like many of the fossil hominin prints, often lost their distinctive toe ridges (**B**) in minutes to hours. Of the 188 tracks recorded, 53% were completely obliterated within one week (in blue; N = 103). The exact duration of the remaining tracks (not included in the figure; N = 85) are unknown and placed at 8–29 days. Two percent of all prints remained recognizable after 1 month. Tracks deteriorated due to sediment slumping and settling, overprinting by other tracks or were washed away by water movement. These data suggest that the many high definition hominin prints at Ileret were formed and buried within the same day, likely within a few hours. Photographs: K. Hatala.

This sedimentary sequence was deposited by relatively low energy fluvial and shallow lacustrine processes on a deltaic lake margin. The fossil tracks are located on multiple discrete, bedded silt layers found throughout the 20ka sequence and on the Ileret Tuff layer itself. Many prints preserve fine detail, such as ridges between the toes, indicative of mud that was both plastic and firm enough to retain shape after the tracks were formed. Typically, tracks were infilled by fine or silty sand prior to deposition of the following silt layer. In some cases this depositional couplet was repeated multiple times. No soil development or root traces occur within the footprint layers, showing these surfaces were quickly buried after the tracks were emplaced. Taphonomic experiments conducted on modern human footprints made on muds along the shores of Lake Turkana show that on average human footprints retain fine detail for 1.3 days (Fig. 3), consistent with data from non-hominin fauna in similar environments^8^. Furthermore, the complete absence of mud cracks on any of the layers indicates a high, stable water table and is strong evidence that most tracks were formed and buried within the same day, perhaps within a few hours.

## Paleoenvironment

The tracks of aquatic taxa such as *Hippopotamus, Crocodylus* and water birds further support the presence of a stable body of water in the immediate vicinity, inferred to be a paleolake to the west of the study site. While local soil isotopes (**δ**^18^O and **δ**^13^C) from the early Pleistocene show increasing aridity consistent with global cooling^24^,^25^,^26^, the presence of a lakeshore in the Ileret area indicates the persistence of the Lorenyang lake and its moderating influence on local environmental conditions^3^,^27^. We infer that regional rainfall in the Ethiopian highlands drained into Lake Turkana via the Omo River 1.5 million years ago, as it does today, stabilizing the paleolake level in an otherwise arid landscape^28^.

The most frequently recovered tracks are those of artiodactyls (Bovidae and Suidae, N = 315). Although some tracks are distinctive enough for generic attribution (e.g. *Syncerus, Tragelaphus*), we conservatively ascribed all to suborder and size class (Supplementary Note 1). At the site of FwJj14E (upper), large numbers of medium sized bovid tracks are oriented to the northwest, along and slightly towards the inferred paleolake margin (N = 54; 45 heading NW). This non-random, directional movement (Rayleigh’s test; *P* < 0.001) suggests these animals traveled together as a herd, potentially towards a foraging or drinking site. Fossil bones and teeth systematically collected in the same area and from this same sedimentary sequence, the Ileret Tuff Complex (ITC), show a large proportion of grazing alcelaphins and reduncins indicating open grasslands. Higher abundances of fossils from browsing bovids collected from contemporaneous ITC deposits approximately 5 km further away from the paleolake indicate a transition to mixed grasslands/shrublands (Supplementary Note 2). Similar conditions are found today bordering modern Lake Turkana, where a zone affected by the high water table of the present-day lake excludes woody rooted trees and brush, allowing only water-adapted grasses and sedges to survive.

Avian tracks are common (N = 29) and consistent with a lakeshore bordered by a grassland environment. Tracks of water birds such as geese (Anatidae) and small wading birds indicate close proximity of surface water to the footprint locales at the time of deposition. Distinctive *Pelecanus* tracks, a taxon only found near large, open bodies of water, strongly suggest that these tracks were made in the transition zone between river delta and paleolake. The tracks of larger wading birds such as storks (Ciconiidae) and cranes (Gruidae), which forage in shallow water and in grasses near water, support the presence of lake margin grasslands. Very large avian tracks, provisionally ascribed to the extinct, giant marabou stork (*Leptoptilos falconeri*), may represent the last known appearance of this taxon^29^ (Supplementary Note 3).

## Hominin Abundance

**Figure 4:**
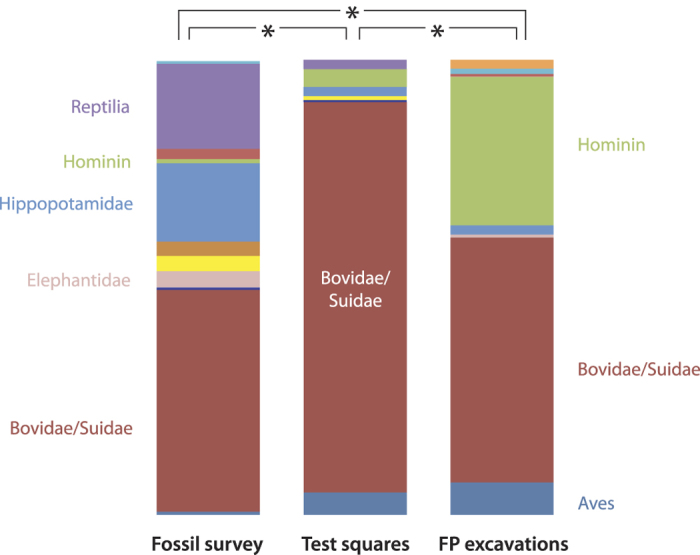
Relative frequencies of tracks and skeletal fossils. Hominins are abundant in both the track excavations (right) and random test squares (center) relative to their scarcity in the skeletal fossil record (left) from the same strata (data in Supplementary Note 6; asterisk = 0.0005 > *P* > 0.00009). Based on these samples, hominin print prevalence indicates frequent and repeated use of lake margin habitats.

To further understand the paleoecological and behavioral significance of the trackway record, we examined how the trackway faunal proportions compares with that in the skeletal fossil record (N = 593). Given that long-term preservation of tracks required rapid deposition, the fossil tracks were non-randomly clustered in areas where such deposition was more likely to occur, e.g., areas of active deposition near the delta or lake margin. This could result in a spatial and environmental bias in the footprint record compared with the skeletal fossil material from the ITC, which was preserved in depositional environments with varying proximity to the lake margin. However, most of the skeletal fossils preserved in the ITC also are from rapidly deposited lithofacies associated with fluvial and deltaic processes. All of the taxa in question were highly mobile and thus should fairly represent the faunal community that had access to lake margin environments. We compare footprint abundance to skeletal fossil frequency as a proxy measure for how frequently an animal used a specific portion of the landscape versus how common that animal was within the overall faunal community. These comparisons show that bird, bovid and hominin tracks are all significantly more abundant than skeletal fossils of these same taxa (Fisher’s exact - Monte Carlo, 0.0005 > *P* > 0.00009; Fig. 4).

While taphonomic biases favoring the preservation of skeletal material from large aquatic animals versus delicate bird bones may explain some of the differences between assemblages, these biases do not account for discrepancies in the hominin and bovid material. An additional potential source of bias stems from the fact that track surfaces can, and often do, preserve multiple tracks from the same individual. These repeated records complicate findings about how many individuals are represented on these surfaces and when and how often those individuals traversed the landscape. While size variation in the tracks, identifiable gait patterns and similar preservational state give us confidence in estimating the minimum number of individuals present and judging group characteristics for hominins, this is more difficult to accomplish for other animal tracks and may alter our analyses of faunal abundance. We have attempted to adjust for repeated printing in our analyses (by correcting for number of track making limbs per individual, estimated stride length, body mass). However, each of these corrections increased relative hominin abundance in our dataset (e.g. an individual bovid makes four tracks per stride compared with the two per stride made by a hominin). We have conservatively chosen to use the uncorrected data. Accordingly, our hominin footprint abundance percentages should be considered minimum estimates. The skeletal and dental fossil data also may include remains from the same individual. This potential bias is minimized using square and bone walk surveys that employ total collection methods, where teeth and/or bone fragments from a single individual are given one specimen number. These data include a consistently small number of hominin remains in samples from the early to late 1970’s^30^.

**Figure 5:**
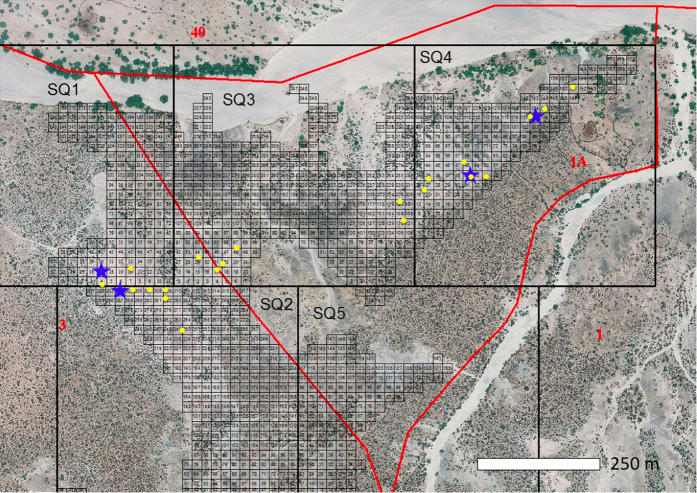
Random sampling of test square locations. Large grid squares (500 m^2^; SQ#) divide the collection areas (bounded in red). Smaller numbered squares overlay exposed Okote Mb. deposits and were used to assemble a sample of unbiased sites. Random numbers were generated in proportion to the number of Okote Mb. sub-units in each large square and surveyed for tracks. If a single track of any species was found within 50 m of the target unit, a test square was gridded and excavated (locations in yellow). 41% of randomly chosen units contained tracks. The distribution of sites reflects the concentration of sediment exposures. Larger targeted excavations are shown with blue stars. Map: ArcGIS version 10.2.

Hominin footprints comprise a third of all tracks recovered from the targeted excavations (N = 89), despite hominin fossils representing <1% of the identifiable skeletal fossil assemblage from the same stratigraphic interval. Given that initial excavations specifically targeted areas where hominin tracks had been found, collection bias inflates their frequency relative to other animal tracks compared with their actual prevalence on the paleolandscape. To address this, georectified aerial photographs of Okote deposits were gridded using ArcGIS, and GPS coordinates were randomly generated to select twenty unbiased 1 m^2^ test squares within the ITC (Fig. 5). Test square print frequencies differed significantly from both targeted footprint excavations and fossil survey data (Fisher’s exact - Monte Carlo, 0.0005 > *P* > 0.00009; Fig. 4). Hominin prints within the random squares (N = 8 tracks) were far less common than in the targeted excavations, but still over four times more abundant than expected based on skeletal fossils from the same deposits.

## Hominin Behavior

Hominins appear to have traversed this lakeshore habitat frequently, in striking contrast to their limited abundance in the local fossil assemblage and inferred scarcity in the living ITC faunal community^30^,^31^. The high probability of finding additional hominin tracks at any site containing at least one other hominin’s prints suggests that their tracks are prevalent because they traveled together. Prints recovered at FwJj14E (upper) preserve toe ridge details, show minimal overlap and have minimal overprinting by other hominin prints in the sub-parallel trackways (1 sub-parallel hominin trackway shows at least one instance of hominin overprinting)^21^. These data strongly support instantaneous emplacement of the tracks by multiple, large-bodied hominins traveling in the same direction (Rayleigh’s test, *P* < 0.001; N = 55). Based on the size, shape similarity and modernity of these prints, they are tentatively attributed to a group of *Homo erectus* individuals, including multiple males^21^.

**Figure 6:**
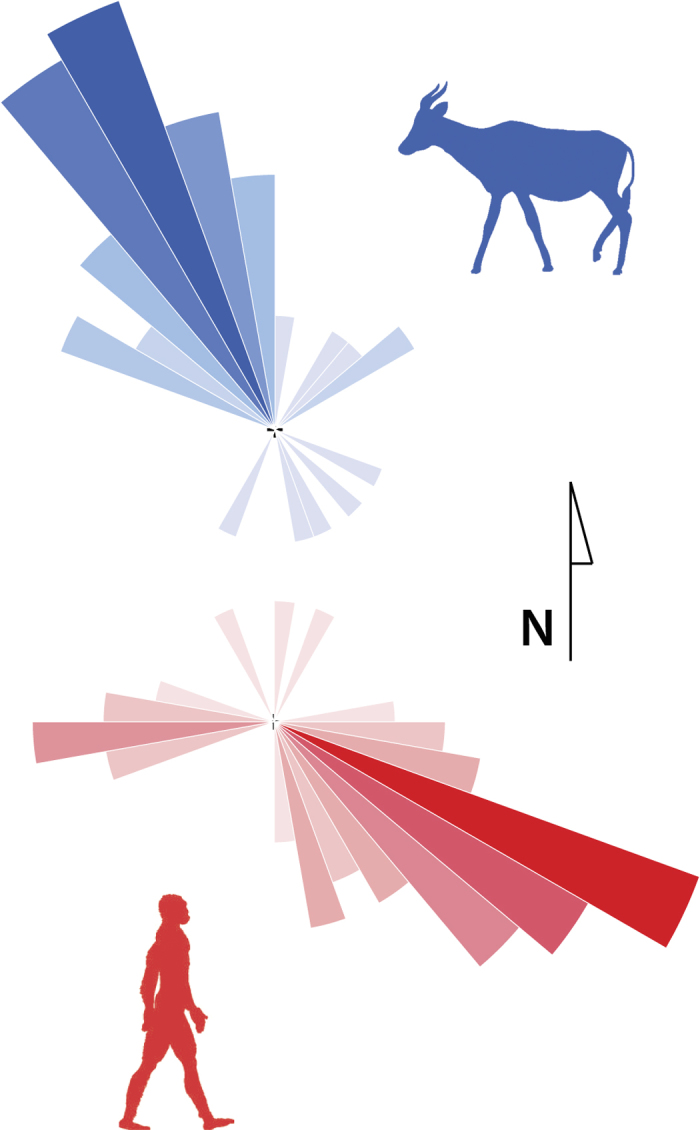
Directional movement patterns from tracks excavated from a single level at FwJj14E (upper). Bovid tracks (blue) are significantly oriented (*P* < 0.0001) to the northwest. Hominin prints (red) are significant oriented (*P* < 0.0001) towards the southeast, generally parallel to the inferred paleolake margin. Shaded sections in both rose diagrams bin individual tracks in 10° increments, with longer, darker sections reflecting more tracks. Silhouettes: www.phylopic.org.

Directionality differences between the bovids and hominins suggest that movement patterns are not physically constrained by landscape features. This is also true within each taxon, as isolated bovid and hominin trackways heading in different directions than the larger groups confirm an open, unconstrained land surface. This reconstruction is further supported by the geology of the ITC, indicating a flat, low-energy deltaic plain bordering a lake. We argue that patterns of movement were more likely affected by the resources the paleolakeshore provided rather than the lake acting as a physical barrier. The southeastern orientation of the hominin tracks suggests that they generally moved along the lakeshore (Fig. 6; Supplementary Note 4). This movement pattern, which follows the land-water ecotone, is an effective way of foraging for nutrient rich plants and animals. It also mirrors movement patterns of modern day carnivores (Supplementary Note 5), which show non-random attraction to, and movement along, fixed waterways^32^,^33^. These carnivore land use patterns likely reflect high prey density around arid landscape water sources^34^,^35^, as well as the scavenging of food that washes ashore, both strategies that reduce search and stalking effort.

Near-water microhabitats may have had a strong selective role in altering the behavior and evolution of *H. erectus.* Although our fossil survey findings and previous faunal analyses indicate that *Homo* was commonly associated with grassland habitats^36^, finer scale ecological data presented here suggest that at 1.5 Ma in the northern Turkana Basin at least some of these grasslands were near water and likely edaphic^36^. Further, oxygen isotope data collected from *H. erectus* teeth show lower δ^18^O values than earlier *Homo*, consistent with better-watered habitat and food^37^. Evidence of hominin consumption of aquatic plants and animals such as fish, turtles and molluscs dates back to at least *H. erectus* and such dietary resources may have contributed to brain expansion in later *Homo*^38^,^39^,^40^. Near-water habitats are also effective places to both hunt and scavenge animals coming to drink. The proliferation of stone tools and cut-marked bone in the fossil record are contemporaneous with the emergence of *H. erectus* and indicative of increased carnivory. Given that the acquisition of meat is both costly and has a high probability of failure (e.g.^41^), reliance on high-quality aquatic foods^42^ and social support^43^ may have been crucial for early hunters.

As the first hominin species to migrate out of Africa, *H. erectus*’ global expansion would have required moving through and surviving in inhospitable environments. Consistent access to water would have allowed *H. erectus* to sweat effectively without dehydrating^44^, increasing day range and mobility. Near-water habitats such as lake margins and rivers may have provided corridors for long distance travel and migration^45^,^46^. These aquatic corridors would have made access to food and water more predictable, buffering hominins from climate change, particularly the increasingly arid conditions in North Africa that our ancestors would have faced as they spread out of the continent.

## Methods

### Taphonomic experiments

To assess the durability and unique preservational conditions of the Ileret fossil tracks, five unshod modern human subjects created footprints (N = 118) on the moist, sub-aerial and shallow water-covered silty muds along the shores of Lake Turkana. The experimental tracks were photographed, measured and their shape parameters were recorded every 6–12 hours over the first 3–7 days following emplacement. Sporadic assessment of remaining tracks occurred for up to 30 days. Tracks were classified as either high definition (ridges between the toes present; Day 0 & 1), recognizable (outline visible, identifiable as a human print; Day 2) or absent (Fig. 3B). All experimental subjects provided informed consent. Subject participation was conducted in accordance with protocols approved by the George Washington University human subjects committee.

### Targeted excavations

Following the initial discovery of hominin footprints at the site of FwJj14E in 2006, a large expanse of the upper track surface (54 m^2^) was gridded and discontinuously excavated (in 1 m^2^ units using 10 cm spits) over a period of 10 years. The sedimentary layers are relatively soft and excavation was done with brushes and small digging tools. In most cases, the tracks could be exposed with gentle brushing, removing infilled sand. When fully exposed, all fossil tracks were identified, catalogued, mapped using a Topcon GTS total station and extensively photographed for 3D photogrammetric modeling. In 2013, three additional sites, two containing hominin prints, were located during survey. These sites (FE 1–8 m^2^; FE 2–14 m^2^; FE 3–18 m^2^) were excavated and recorded using the same procedures. Track orientations were trigonometrically calculated using R software (v. 2.15.2) from Cartesian coordinates collected at the anterior and posterior midline of each track and converted to global magnetic orientations using known track orientations measured with a Brunton compass.

### Fossil surveys

Fossil data were systemically collected in the 1970’s by AKB and colleagues from within the same stratigraphic interval (between the Northern and Lower Ileret tuffs). Fossil surveys used total collection methods in which all identifiable bones and teeth observed in the survey area were recorded. Data from Area 1 A (where the tracks are located) include 10 m^2^ surface “squares” recorded in 1970–72, as well as “bone walk” data collected in 1979^30^. The latter are careful surveys that document all surface fossils associated with specified stratigraphic intervals between the marker tuffs or other distinctive beds. Faunal comparison to Areas 8 and 8 A also use squares^47^ and bone walk data collected in 1979. These older datasets helped to minimize the biasing effects of selective collection of hominin, primate and other rare taxa since that time. Data were pooled by collection area as no statistical differences were found between survey modalities.

### Random test squares

In 2014, twenty 1 m^2^ test squares were excavated within the ITC in Areas 1 A and 3. These test square excavations were designed to remove the effects of collection and spatial bias in the track data by randomly sampling exposed ITC sediments in the collection areas. To implement the random sampling, georectified aerial photos of Areas 1 A and 3 were fitted with 500 m^2^ squares in ArcGIS (v. 10.2). A series of numbered 20 m^2^ squares were then placed in each larger square, overlaying only exposed Okote Member deposits (Fig. 4). A random number generator algorithm (Excel v. 14.4.8) was then used to select potential test square sites. GPS coordinates for these randomly selected units were taken from the georectified photographs and used to target their location. If intensive surface survey within 50 m of the targeted square yielded at least 1 identifiable track from any taxon, a 1 m^2^ test unit was gridded and excavated. All tracks within the test squares were then mapped, measured and extensively photographed.

### Statistical treatment

Comparisons of track and skeletal fossil frequencies were conducted using Fisher’s exact test^48^ due to small sample sizes in some taxonomic groups. Given that *P* values are not exact for contingency tables larger than 2 × 2^49^, *P* was estimated using Monte Carlo approximation methods with 2000/10,000 iterations. Directionality in track assemblages was assessed using Rayleigh’s test of uniformity for circular data^50^. All statistical analyses were conducted using R software (v. 2.15.2).

## Additional Information

**How to cite this article**: Roach, N. T. *et al*. Pleistocene footprints show intensive use of lake margin habitats by *Homo erectus* groups. *Sci. Rep.* **6**, 26374; doi: 10.1038/srep26374 (2016).

## Supplementary Material

Supplementary Information
