# Supplementary material for: Pleistocene footprints show intensive use of lake margin habitats by Homo erectus groups
Source: Sci Rep. 2016 May 20;6:26374. doi: 10.1038/srep26374 (PMC4873780; doi:10.1038/srep26374)
Supplement: Supplementary Information [file srep26374-s1.pdf]

Supplementary Material for

Pleistocene footprints show intensive use of lake margin habitats by *Homo erectus* groups

Neil T. Roach *, Kevin G. Hatala, Kelly R. Ostrofsky, Brian Villmoare, Jonathan S. Reeves, Andrew Du, David R. Braun, John W. K. Harris, Anna K. Behrensmeyer, Brian G. Richmond

*Correspondence to: ntroach@fas.harvard.edu

This file includes:

Supplementary note 1	Bovid/suid frequency by size class
Supplementary note 2	Bovid taxic proportions (Areas 1A and 8A)
Supplementary note 3	Tracks identified as *Leptoptilos falconeri*
Supplementary note 4	Directional movement patterns of hominin tracks (FE 3)
Supplementary note 5	Near-water land use by modern fauna
Supplementary note 6	Identifiable skeletal fossil and track counts

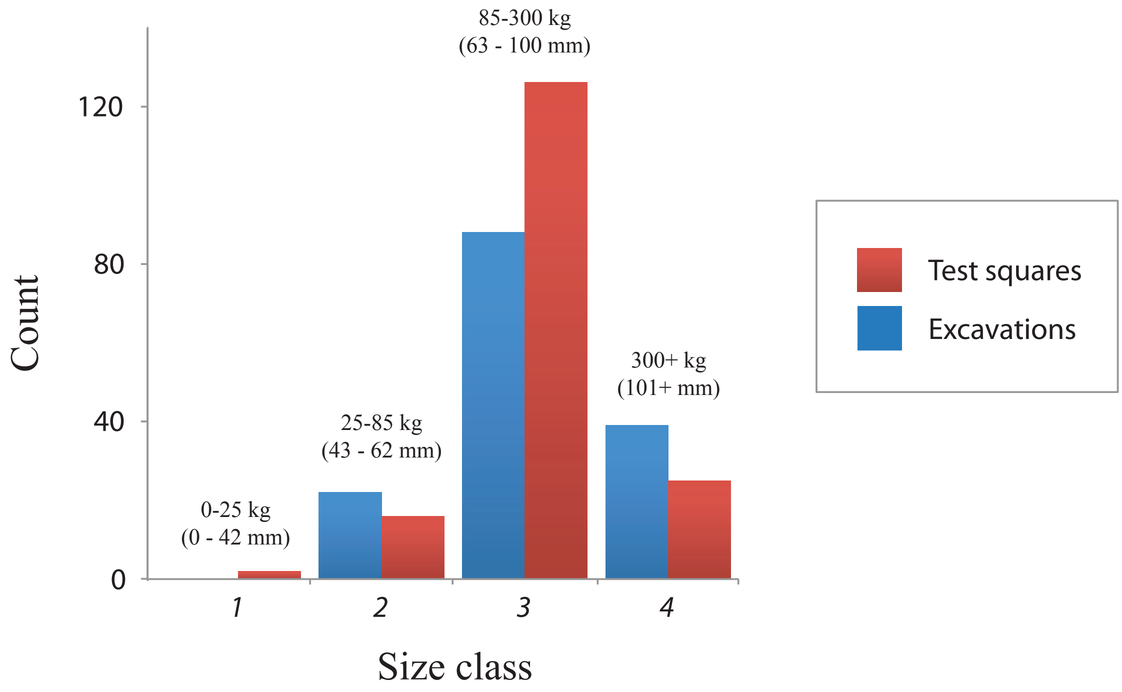

Supplementary Note 1 | *Bovid-suid track frequency by size class.* Most cloven hooved tracks recovered are not distinctive enough for species attribution (Fig. S1). These tracks were instead separated into size classes. Track size classes were adapted from Brain's bovid classification scheme [52] and converted to track length using maximum dimensions for representative fauna [53]. Medium sized bovids/suids (e.g. wildebeest or waterbuck size) predominate in both the targeted excavations and random test squares.

Supplementary Note 2 | *Bovid taxic proportions (Area 1A and 8A)* – Area 1A, where the track sites are located, contains a largely grazing bovid fauna (high percentages of alcelaphins and reduncins)(Table S1). These data are consistent with a more open, grasslands habitat. Area 8A, located approximately 5km further away from paleo-Lake Turkana, had a more diverse and mixed bovid fauna with fewer pure grazers and more browsers/mixed feeders (e.g. aepycerotins and tragelaphins). These differences in taxic composition are significant (Fisher's exact - Monte Carlo, $0.0005>P>0.00009$), however sample size effects may alter overall diversity and increase significance levels. Preservation conditions (% of recorded specimens that are identifiable) are similar between collection areas.

Tribe	Area 1A	Area 8A
Aepycerotini	4.3	17.9
Alcelaphini	43.5	29.1
Antilopini	0	4.3
Bovini	8.7	7.7
Hippotragini	0	0.9
Reduncini	39.1	29.9
Tragelaphini	4.3	10.3
**N**	**Area 1A**	**Area 8A**
Identified	23	117

Table S1 | Bovid taxic proportions (percentage of overall bovid assemblage) based on systematic surveys of skeletal remains in Areas 1A and 8A.

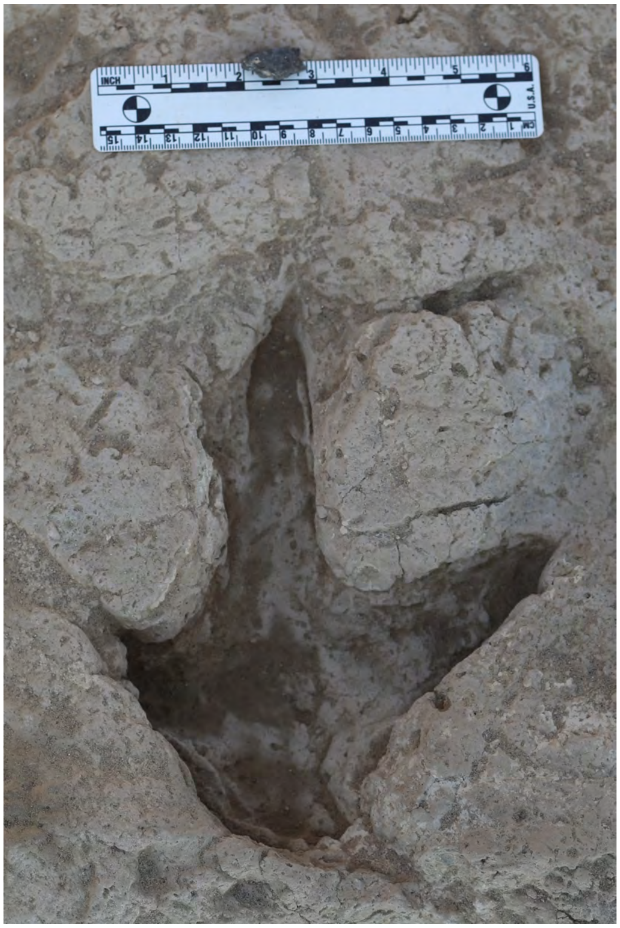

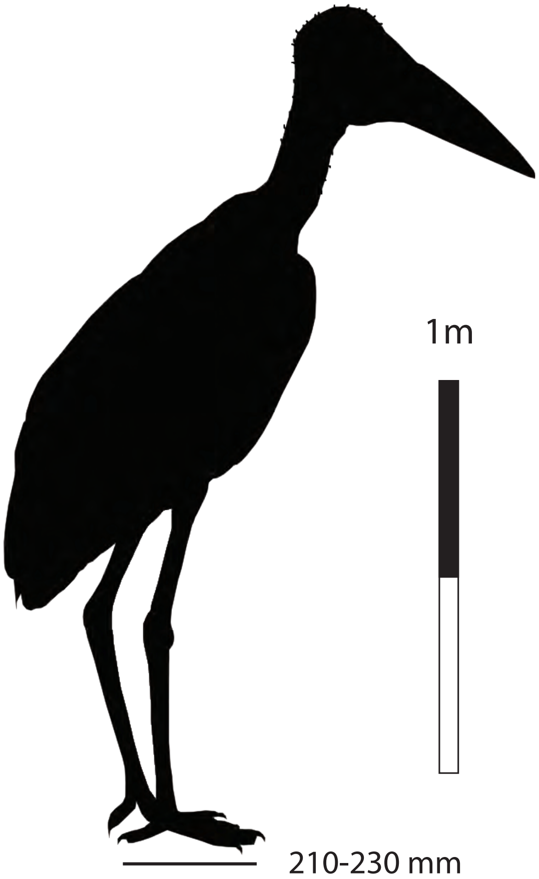

Supplementary Note 3 | *Tracks identified as Leptoptilos falconeri*. Four very large avian tracks were recovered at random test square FE 18. These tracks resemble those of a tall wading bird, showing 3 ventral facing digits and a dorsally-oriented hallux (Fig. S2). However, these tracks dwarf those of all extant African birds (track lengths reach 215mm and span up to 230mm). The size and shape of these large avian tracks is consistent with that of the extinct 2m tall marabou stork *Leptoptilos falconeri,* which was 15-20% larger than the extant marabou species, *L. crumeniferus* (tracks can reach 195mm) [54]. While the age and geographic range of this species is uncertain [55,56], it is currently thought to have gone extinct in the late Pliocene. These tracks would significantly extend the last appearance datum of this giant stork. Photograph: K. Hatala.

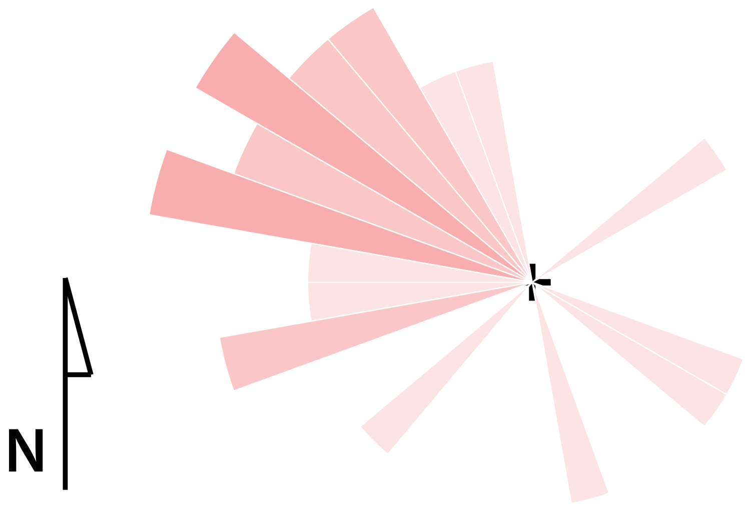

Supplementary Note 4 | *Directional movement patterns of hominin tracks (FE 3)* - The only trackway site other than FwJj14E with sufficient sample sizes to address directionality is FE3, which is lower in the stratigraphic sequence. Hominin prints from this site (N=23) show statistically significant directional movement of multiple, large-bodied individuals to the northwest (*P*=0.0002; Fig. S3). These data are consistent with movement along the paleo-lakeshore by a multi-male group. Note, both the modern lakeshore near Ileret and reconstructed high-stand paleolake (Lorenyang) have a shoreline oriented northwest/southeast [57]. The bovid tracks from this site show no orientation patterning (N=42; *P*=0.5451), suggesting that landscape features do not constrain travel direction on this track surface.

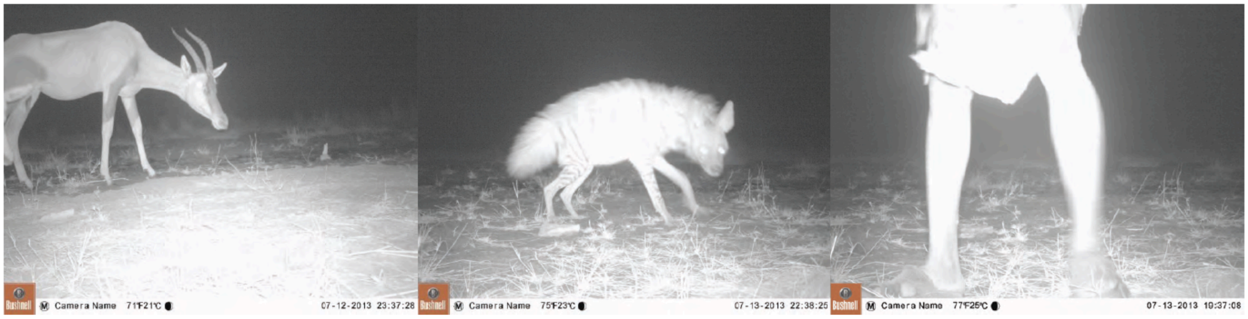

Supplementary Note 5 | ***Near-water land use by modern fauna*** - Motion activated cameras were used to monitor animal activity at the track taphonomy experimental sites along the shore of Lake Turkana. Here, we show representative night image captures of: L to R; a topi (*Damaliscus korrigum*), striped hyena (*Hyaena hyaena*) and Daasanach fisherman (Fig. S4). Birds were most frequently photographed, but showed no general directional land use patterns. The bovids in our experimental data (topi and zebu domestic cattle) typically moved perpendicular to the lakeshore, from the grassy floodplains to the water and back. This land use pattern may differ in taxa adapted to an edaphic grazing diet. Note: The cattle did occasionally travel parallel to the lakeshore when actively shepherded by human minders. Carnivores (black-backed jackals, striped and spotted hyenas) typically moved parallel to the waterline, travelling up and down the lakeshore. Modern human land use patterns along the lakeshore match carnivore patterns, as Daasanach men and children forage and fish along the lake. Photographs: K. Hatala/N. Roach.

Supplementary Note 6 | *Raw counts of identifiable skeletal fossil and tracks* – Numbers of tracks in parentheses denote some uncertainty in identification. In the stacked bars in Fig. 4, the order of the taxonomic categories is reversed.

**Taxon**	**Skeletal Fossils**	**Footprint test squares**	**Footprint excavations**
Aves	1	10	19
Bovidae/Suidae	293	176	149
Carnivora	2	1	(1)
Elephantidae	21	0	1
Equidae	21	2	0
Giraffidae	18	0	0
Hippopotamid	105	4	9
Hominin	2	8	89
Primates	14	0	(1)
Reptilia	113	4	0
Rhinocerotidae	3	0	2
Tubulidentata	0	0	(5)
**N**	**593**	**205**	**276**

References:

52 Brain, C. K. Some suggested procedures in the analysis of bone accumlations from southern African Quaternary sites. *Annls Transv Mus* **14**, 1-8 (1974).

53 Stuart, C. & Stuart, T. *A field guide to the tracks & signs of Southern and East African wildlife*. 3rd edn, (Struik Publishers, 2000).

54 Milne-Edwards, A. *Recherches anatomiques et paléontologiques pour servir à l'histoire des oiseaux fossiles de la France*. Vol. II (E. Martinet, 1868).

55 Louchart, A., Vignaud, P., Likius, A., Brunet, M. & White, T. D. A large extinct marabou stork in African Pliocene hominid sites, and a review of the fossil species of Leptoptilos. *Acta Palaeontol Pol* **50**, 549-563 (2005).

56 Hill, A. & Walker, A. A fossil marabou (Aves: *Ciconiidae*) from the Miocene Ngorora formation, Baringo district, Kenya. *Neth J Zool* **29**, 215-220 (1979).

57 Feibel, C. S. A geological history of the Turkana Basin. *Evol Anthropol* **20**, 206-216 (2011).
